# β-Amyloid and the Pathomechanisms of Alzheimer’s Disease: A Comprehensive View

**DOI:** 10.3390/molecules22101692

**Published:** 2017-10-10

**Authors:** Botond Penke, Ferenc Bogár, Lívia Fülöp

**Affiliations:** 1 Department of Medical Chemistry, University of Szeged, H-6720 Szeged, Dóm Square 8, Hungary; fulop.livia@med.u-szeged.hu; 2 MTA-SZTE Biomimetic Systems Research Group and Department of Medical Chemistry, University of Szeged, H-6720 Szeged, Dóm Square 8, Hungary; bogar@sol.cc.u-szeged.hu

**Keywords:** Alzheimer’s disease, protein and lipid dyshomeostasis, APP, intracellular Aβ, ER-mitochondrial axis, Ca^2+^ dysregulation, molecular chaperones, autophagy, neuroinflammation, Aβ-clearance

## Abstract

Protein dyshomeostasis is the common mechanism of neurodegenerative diseases such as Alzheimer’s disease (AD). Aging is the key risk factor, as the capacity of the proteostasis network declines during aging. Different cellular stress conditions result in the up-regulation of the neurotrophic, neuroprotective amyloid precursor protein (APP). Enzymatic processing of APP may result in formation of toxic Aβ aggregates (β-amyloids). Protein folding is the basis of life and death. Intracellular Aβ affects the function of subcellular organelles by disturbing the endoplasmic reticulum-mitochondria cross-talk and causing severe Ca^2+^-dysregulation and lipid dyshomeostasis. The extensive and complex network of proteostasis declines during aging and is not able to maintain the balance between production and disposal of proteins. The effectivity of cellular pathways that safeguard cells against proteotoxic stress (molecular chaperones, aggresomes, the ubiquitin-proteasome system, autophagy) declines with age. Chronic cerebral hypoperfusion causes dysfunction of the blood-brain barrier (BBB), and thus the Aβ-clearance from brain-to-blood decreases. Microglia-mediated clearance of Aβ also declines, Aβ accumulates in the brain and causes neuroinflammation. Recognition of the above mentioned complex pathogenesis pathway resulted in novel drug targets in AD research.

## 1. An Overview of the Possible Pathomechanisms of Alzheimer’s Disease (AD)

Nascent protein chains, emerging from the ribosomes, need to fold properly into unique 3D structures, eventually translocate and then assemble into stable, functionally flexible complexes [1]. In the crowded cellular environment, newly synthesized polypeptide chains are at risk of misfolding, forming stable, toxic aggregates. These species may accumulate in aged animal cells, especially in neurons and can cause cellular damage inducing cell death [2]. Aggregation of specific proteins into protein inclusions and plaques is characteristic for many neurodegenerative diseases (NDDs), including AD, Parkinson’s (PD), Huntington’s disease (HD) and amyotrophic lateral sclerosis (ALS). Table 1 summarizes the short list of some NDDs and their corresponding misfolded proteins [3].

Molecular pathological classification of neurodegenerative diseases is based on the presence of these pathologically altered, misfolded proteins in the brain as deposits [4]. These proteins and their biochemical modifications can potentially be targeted for therapy or used as biomarkers (e.g., CSF-biomarkers in AD [5]) sometimes combined with neuroimaging modalities [6,7]. The combination of proteinopathies is also frequent.

What is the mechanism of formation of toxic protein aggregates in a living cell? Proteins are structurally dynamic and thus constant surveillance of the proteome by integrated networks of chaperones and protein degradation machineries (including several forms of autophagy) are required to maintain protein homeostasis (proteostasis) [1]. NDDs are considered mostly as pathologies of disturbed protein homeostasis. Proteostasis network declines during aging, triggering neurodegeneration and other chronic diseases associated with toxic protein aggregation [8]. In both aging and AD there is a general decrease in the capacity of the body to eliminate toxic compounds. In AD, toxic β-amyloid (Aβ) and hyperphosphorylated Tau (pTau) aggregates may interact with subcellular organelles of the neurons, trigger neuronal dysfunction and apoptosis that lead to memory decline and dementia.

Several studies demonstrate the central role of disturbed protein homeostasis in the pathogenesis of AD. Transcriptional signature of AD is associated with a metastable subproteome at risk for aggregation [9]. A proteostasis signature in healthy brain recapitulates tissue vulnerability to AD [10]: neurons contain suboptimal levels of protein homeostasis components (low levels of aggregation protectors and presence of aggregation promoters).

Aging *per se* is the most important factor of AD and several other neurodegenerative diseases, “the neurobiology of aging and AD is walking down the same road” [11]. AD could be seen as a “maladaptive interaction between human brain evolution and senescence” [12]. Other authors hypothesized that formation of aggregated proteins might be a protective strategy of the aging neurons [13]. Very recent results demonstrate that transposon-mediated genomic instability plays a key role in the aging process [14].

There are numerous hypotheses for understanding the pathogenesis of AD, owing to the multifactorial character of the disease. Some of them (disturbance of the cholinergic system; hypoperfusion, hypoxia in the brain; Ca^2+^-signalization problems; neuroinflammation; mitochondrial dyshomeostasis; chronic ER-stress and protein misfolding; decreased Aβ-clearance, etc.) are not controversial and could be unified into a general broad hypothesis. The common nominator of these hypotheses is the important role of Aβ in the pathogenesis of AD.

The conventional view of AD is that much of the AD-pathology is driven by an increased load of Aβ in the brain of patients (“amyloid hypothesis” [15]). During the last 15 years many therapeutic strategies were based on lowering Aβ in the brain. Up to now, most of the strategies have failed in clinical trials and the relevance of the amyloid hypothesis has often been questioned [16]. Very recent results show that pathophysiological changes begin many years before clinical manifestation of AD and the disease is a multifaceted process [17]. A rare mutation in the Iceland population gave a strong evidence for the important role of Aβ in the pathomechanism of AD [18]. The core of the amyloid hypothesis stays on and novel clinical trial strategies may hold promise [19].

In the present review article, we summarize the physiological functions of amyloid precursor protein (APP) and the role of amyloid fragments in adult brain. Then we give a short summary on the genetic background of AD, the interaction of Aβ peptides with subcellular organelles, the pathways of Aβ clearance from the brain, the role of neuroinflammation, brain circulation and the blood-brain-barrier (BBB) in the AD pathogenesis. Finally, we discuss very shortly the major trends in drug discovery and the possibilities for prevention and treatment of AD.

## 2. Physiological Functions of the Amyloid Precursor Protein (APP) and Its Metabolites (Amyloid Fragments)

**Figure 1:**
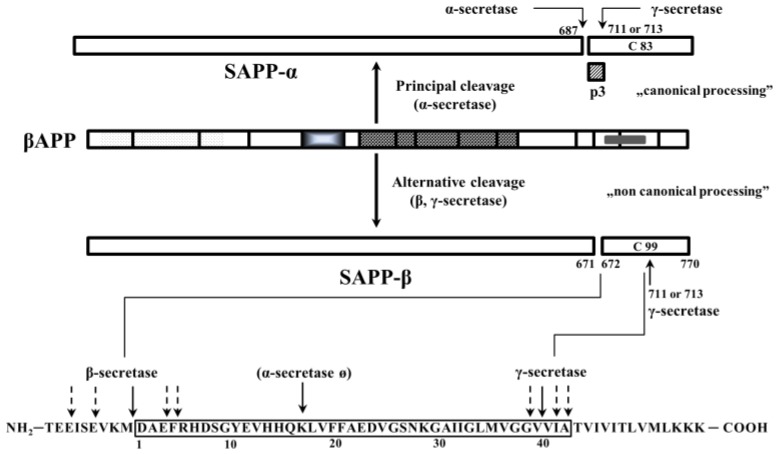
Simplified presentation of the amyloid precursor protein (APP) and its processing products. Principal cleavage of APP with α-secretase results in the formation of water soluble products (SAPP-α, C83 and P3). Alternative (amyloidogenic) cleavage with β- and γ-secretase gives a heterogeneous mixture of β-amyloid (Aβ) peptides of 37 to 43 amino acids.

APP is a transmembrane protein with a large extracellular N-terminal domain, a transmembrane domain and a short C-terminal cytoplasmic domain consisting of 59 amino acid residues [20]. There are eight isoforms of APP; the shortest 695 amino acid isoform is highly expressed in the CNS. Studies on APP overexpression demonstrate that it positively modulates cell survival and growth [21]. APP promotes neurite arborization in a *Drosophila* model of brain injury [22]. APP also plays an important role in the formation and maintenance of synapses, neuronal survival and neuritic outgrowth [23,24,25]. The importance and the functional neurophysiology of the APP-processing pathways and products are widely reviewed by Randall et al. [26]. It is known that APP has two main processing pathways: (1) the canonical, non-amyloidogenic (90%) involving α-secretase and releasing a truncated form of APP (soluble APPα, SAPPα) and the C-terminal fragment C-83; and (2) the non-canonical, amyloidogenic pathway (10%), which generates Aβ peptides through the sequential cleavage by β-secretase (BACE) and γ-secretase (Figure 1).

It was suggested that the non-amyloidogenic cleavage of APP is localized mostly in the plasma membrane [27] and the amyloidogenic cleavage runs intracellularly. After the biosynthesis of APP, the polypeptide chain is transported to the Golgi apparatus, where it is *O*- and *N*-glycosylated, phosphorylated and sulfonated at tyrosine residues [28]. Only 10% of APP goes to the plasma membrane and the majority of APP remains in the Golgi and trans-Golgi network (TGN) according to in vitro studies. APP inserted into the plasma membrane can be internalized via endocytosis (due to the presence of “YENPTY” motif) and reaches the endosome [29]. Several subcellular compartments (trans-Golgi network, lysosomes, and ER-MAM) may participate in APP processing in the cells:

1. Late Golgi compartments could be mainly involved in the generation of intracellular Aβ (iAβ) [30].
2. Recent studies have demonstrated that APP is rapidly transported from the Golgi apparatus to the lysosome, where it is processed into Aβ [31]. The adaptor protein 3 (AP-3) mediates rapid delivery of APP to lysosomes.
3. Several studies demonstrated, however, that α- and β-secretases are present in ER allowing APP processing [32,33]. It was shown that presenilin 1 and 2 (PSN1, PSN2) and γ-secretase activity are located in the special subcompartment of the ER, called mitochondrial associated membrane (MAM). It was hypothesized that MAM is a subcellular site of the amyloidogenic processing of APP and Aβ generation [34,35]. Most recent results demonstrate that vascular dysfunctions, exposures to hypoxia/ischemia increase the amyloidogenic processing of APP and provoke AD pathology by activating β- and γ-secretases [19].

Up to now, most APP-related research has focused on the amyloidogenic properties and toxicity of Aβ peptides, with less emphasis on the normal physiological roles of APP and its other cleavage products. APP family (APP and APP-like proteins) remained a biochemical enigma in brain development and function [36]. Novel studies on the physiological functions of the protein have opened insight into the role of APP beyond AD, emphasizing the neuroprotective role of APP and its metabolites [37]. The short summary of the most important new results are:

1. APP plays a key role in neuronal homeostasis [38] including intracellular transport and signaling. APP acts as a signaling connection that transduces information about extracellular conditions (e.g., neuronal damage) and induces intracellular signaling events. Minute disruptions in APP signaling functions may be major contributors to neuronal dysfunction leading to AD.
2. APP has a trophic function and may be involved in neuronal stem cell development, neuronal survival, neurite outgrowth and neurorepair [21,22,37].
3. Most recent studies proved the results on the key role of APP as a neuroprotective factor [39]. Acute (stroke, cardiac arrest) or chronic (cerebrovascular disease) hypoxic-ischemic conditions cause up-regulation of APP. The protein itself and its soluble extracellular fragment SAPPα can promote neuronal survival. The underlying mechanism is very probably APP-mediated regulation of calcium homeostasis.
4. Among the three major splice isoforms of APP (APP_695_, APP_751_, APP_770_), the predominant neuronal form is APP_695_. Beyond these, closely related APP-like proteins (APLP1, APLP2) exist as members of the APP family. All the APP family members are truly multifunctional proteins and can form large signaling complexes with various transmembrane proteins and intracellular binding partners [40].
5. Canonical and non-canonical processing of APP results in a series of APP fragments, the most important fragments are SAPPα, SAPPβ, Aβ (40 and 42 AA length) and AICD. Their in vivo functions in the CNS are widely reviewed by Müller et al. [40], here we give a very short summary of their most important role:
  SAPPα:
    rescues memory and spine density in aged rats
    reduces plaque deposition and tau phosphorylation
    protects against traumatic brain injury (TBI), neuronal death in transient ischemia and hypoxia
    stimulates adult neurogenesis
  AICD:
    modulator of gene expression apoptosis and cytoskeletal dynamics [41]
    indirect regulation of DNA damage response
  Aβ 40/42:
    regulation of lipid homeostasis [42].
    regulates neuronal homeostasis in picomolar amounts (stimulating PTP, LTP and memory)
    when their production and degradation are imbalanced, the peptide forms toxic Aβ-assemblies in higher concentration [43,44].

In summary, it can be stated that APP and its metabolites (SAPPα, AICD, native Aβ) have important physiological functions in cell mainly in normal neuronal development, neurogenesis, neuronal homeostasis and different signalization pathways.

## 3. Formation and Propagation of Hypertoxic Aβ Structures and the Prionoid Hypothesis

Emerging evidence supports the hypothesis that Aβ monomers in picomolar level possess neuroprotective activity [45]. However, understanding the role of Aβ in the neurodegenerative process of the disease has been the central challenge. The continually (sometimes radically) changing amyloid cascade theory hypothesized a seeding-nucleation model for Aβ aggregation [46] and speculated the following toxic assemblies as causative factors for AD [47]:

1. Fibrillar Aβ
2. Soluble, oligomeric Aβ: Aβ dimers; Aβ trimer, tetramer, hexamer; Aβ dodecamer (56kD, Aβ*56); large Aβ oligomers (“high-n” oligomers)
3. Protofibrils
4. Dispersible Aβ oligomers, protofibrils and fibrils [48,49].

A glossary of Aβ-aggregation products and precise descriptions for preparing different Aβ species are given by Jan et al. [49]. What is the toxic or most toxic Aβ-assembly and what is the role of Aβ in AD? These questions have remained the subject of hot debates [50,51,52]. Analysis of the soluble and insoluble amyloid material of human AD brain demonstrated the extraordinary heterogeneity of Aβ peptides (26 proteoforms of different peptide chain length including various N-and C-terminal transactions) [53]. The vast majority of Aβ peptides deposited in AD brains is truncated and post translationally modified at the C-terminus.

Not only might the size of Aβ-aggregates be important for toxicity, but also the peptide conformation. A formidable experimental challenge is the analysis of the biophysical properties and conformation of a single Aβ-species (e.g., Aβ 1-42), mainly because of their nanoscale dimensions and heterogeneous nature [54]. Bulk techniques (CD or IR spectroscopy) are not able to characterize the heterogeneity and inner conformational properties of amyloid aggregates at the single species level.

The fundamental mechanism of protein folding allows the formation of misfolded protein structures [55,56] leading to many fatal diseases. Protein aggregation is regarded as a side reaction of folding. The various mechanisms of protein homeostasis (folding, unfolding, misfolding, aggregation) are summarized in a paper of Goloubinoff et al. [57]). Cells have different mechanisms to deal with protein misfolding and aggregation, where the molecular chaperone machinery constitutes the first line of defence against misfolded proteins. A misfolded protein can be refolded, degraded or aggregated—the last process may lead to toxic assemblies. Aggregation-prone proteins are constitutively expressed in the cell, creating a chronic stress situation. Enhancers and suppressors of aggregation appear during aging and disease [58] for regulating amyloid formation.

Table 1 listed misfolded proteins (similar to Aβ) as causative agents of protein misfolding diseases. The toxic conformational transition would be a therapeutic target for polyQ diseases and probably for conformational diseases in general [59]. Misfolding of α-synuclein is a well-known causative process of PD and LBD [60,61,62]. The spongiform change of the brain in dementia with Lewy bodies and AD connects these disorders to prion diseases [63]. It is assumed that toxic amyloid forming monomers show intrinsically disordered (ID) nature [64,65,66,67].

Aβ may be directly toxic to neuronal cells and synapses. The ID structures of Aβ represent transient intermediates in the aggregation cascade of patient brains. Infectious amyloid material can be generated and this fact bind AD to prion disease [68]. The pathology of AD, the Braak-stages also support the idea that AD spreads from neuron to neuron. It is almost forgotten that at the beginning of the eighties-nineties, AD was suspected of being a prion disease [69]. Recently, it was demonstrated that extracts containing soluble Aβ aggregates induce amyloidosis in mice that otherwise never develop amyloid plaques. Pyroglutamylated Aβ 3-42 shows prion-like behavior in mice [70]. Pathological similarities between AD and prion diseases suggest that the formation and spread of the proteinaceous lesions might involve a common molecular mechanism—corruptive protein templating [71,72,73]. Experimentally, cerebral β-amyloidosis can be exogenously induced by exposure to dilute brain extracts containing aggregated Aβ seeds (seeding-nucleation model). Stohr et al. (in cooperation with Prusiner) [74] have shown that pure Aβ injections into the brain may induce plaques throughout the whole brain within 5 to 6 months. If the peptides were injected into one hemisphere, plaque formation started in both halves of the brain. The amyloid-inducing agent is probably Aβ itself [75], in a special toxic conformation (synonyms: “hypertoxic Aβ”, “primordial cytotoxic Aβ”, “prionoid Aβ”) generated most effectively in the living brain [76]. Amyloid aggregates of one protein are able to directly nucleate amyloid formation of another, different protein: this is called amyloid cross-interaction [77]. Aβ and α-synuclein proteins (associated with AD and synuclopathies) share similar biophysical and biochemical properties with PrP^sc^ that influences how they misfold, aggregate and propagate in disease [78].

The term “prionoid” was introduced by Aguzzi [79] for self-propagating, transmissible protein aggregates [79,80]. Infectious prions and prionoids can be prepared in the laboratory by protein misfolding cyclic amplification (PMCA; [81,82,83,84,85]). The precise molecular mechanism of the conversion of a non-transmissible protein molecule to the pathogenic form (e.g., PrP^c^→PrP^Sc^) is not completely understood. The available data support the seeding-nucleation model in which the infectious conformer (e.g., PrP^Sc^) is an oligomer that acts as a seed to bind native protein (PrP^c^) and catalyze its conversion into the misfolded form by incorporating into the growing polymer [81,86]. Nath et al. [87] demonstrated the transmission of oligomeric Aβ 1-42 after microinjection into primary hippocampal rat neurons via direct neuron-to-neuron transfer. This Aβ-transfer depends on direct cellular connections. As the transferred oligomers accumulate, acceptor cells gradually show beading of tubulin (a sign of neurite damage) and gradual endosomal leakage (a sign of cytotoxicity). This observation supports that intracellular Aβ (iAβ) oligomers play a role in neurodegeneration and explain the progression of AD via anatomical connections.

Very recently Condello and Stohr [88] reviewed novel studies on the prionoid character of Aβ. The progressive nature of AD occurs (at least in part) by the self-replication and spreading of Aβ (and tau) aggregates through a prion mechanism. Evidence exists that structural variants of Aβ-prions can propagate their distinct conformation through template—directed folding of native Aβ peptides. As a consequence, the first self-propagating Aβ assembly emerged in the brain, dictates the conformation, anatomical spread and pace of subsequently formed amyloid deposits. It is hypothesized that the existence of diverse clinicopathological phenotypes (observed among AD patients) might be due to different Aβ strains propagated by prion mechanism. According to Qiang et al. the heterogeneity of AD may be the result of diverse Aβ strains. Structural variation in Aβ-fibrils in AD clinical subtypes indicates that there are structural variations (qualitative differences) between Aβ 1-40 and Aβ 1-42 aggregates in the brain tissue of patients with different AD subtypes of r-AD (rapidly progressive) and t-AD (typical prolonged duration) form [89].

It has remained unclear whether further molecules (co-factors), beside the misfolded prion/prionoid protein, are necessary elements of the infectious agents. Such a co-factor might act as an essential catalyst for prion/prionoid replication, help to stabilize the hypertoxic conformation and increase the biological stability of prions/prionoids. Theoretically, all PMDs have the intrinsic potential to be transmissible [86,90]. Several studies demonstrated Tau-protein transmission [91,92,93,94,95,96]. Tau and Aβ are probably ready for admission to the prion club [97]. Despite many similarities, there is a difference between prion and prionoid diseases: unlike the classical prions, the prionoids (Aβ, Tau, α-synuclein, etc.) do not appear to spread infections from one individual to another. There is still no evidence that AD, PD or ALS are infectious under everyday circumstances.

In summary, it can be stated that in the brain Aβ is a very heterogeneous mixture of peptide with a broad range of aggregation grade and conformational variability. Aβ has very probably prionoid character.

## 4. Short View of the Genetic Background of AD

Although advanced age is the best known risk factor of AD, some individuals may develop AD at a younger age. Hence, based on the time of onset, AD is classified into two types [98]. Early-onset AD (EOAD) typically develops before the age of 65 years, the other form, late-onset AD (LOAD) develops in patients older than 65 years. The production and clearance of Aβ is regulated by a large group of genes. The genetic background of AD is widely reviewed [99,100,101,102]; for references see also http://www.molgen.ua.ac.be/admutations.

EOAD is caused by rare and mostly autosomal dominantly inherited mutations in APP, PSEN1 and PSEN2. These three genes (APP in chromosome 21q, PSEN1 in 14q and PSEN2 in 1q) are considered the main risk factors for EOAD. The mutations within these three genes affect a common pathogenic pathway in APP synthesis and processing (proteolysis), which lead to excessive production of Aβ. To date, more than 270 highly penetrant mutations have been described in these genes that cause familial AD and many more are discovered each year [103]. To date, 49 APP mutations in 119 families are known to cause AD, most of them are dominantly inherited, but two recessive mutations (A673V and E693V) were also demonstrated to cause AD. Most of the pathogenic APP mutations are either adjacent to or within the cleavage sites for β-and γ-secretase, showing that Aβ formation may be involved in AD pathogenesis [104]. A protective mutation of APP against AD (A673T) was also found in the Iceland population [18].

To date, 215 pathogenic mutations of PSEN1 and 13 mutations of PSEN2 have been identified. These proteins are the catalytically active components of γ-secretase. Mutations in PSEN1 account for up to 50% of EOAD with complete penetrance and early age of onset. Mutations of both PSEN1 and PSEN2 alter γ-secretase activity resulting in an increase in the Aβ 42/40 ratio (studies have shown that Aβ 1-42 is more amyloidogenic and prone to aggregate than Aβ 1-40 in the brain [105]).

Strikingly, very recent studies show that only 5% of EOAD patients are carrying a pathogenic mutation in one of the AD genes (APP/PSEN1/PSEN2) or an apolipoproteinE (ApoE) risk allele ε4 [106]. LOAD is genetically far more complex than EOAD with the possible involvement of multiple genes and environmental factors. It is likely that many genes are involved in LOAD pathogenesis, each of small effect and unknown transmission and expression. Most LOAD cases are sporadic with no family history of the disease. Before the era of large scale genome-wide association studies (GWAS), the APOE ε4 allele was the only well-established risk factor for LOAD. During the last 6-8 years of the GWAS era, researchers have identified a number of genes that may increase a person’s risk for LOAD to varying degrees. GWASs identified many common gene variants with low penetrance. It was very interesting that most of the genes identified by GWASs could be linked with the Aβ cascade or Tau pathology. Most of the important 27 genes involved in LOAD pathogenesis can be clustered within three pathways [102], and some genes participate in two pathways:

1. Lipid metabolism:
APOESORL1ABCA7DSG2CLU
2. Inflammatory response. Neuroinflammation is a pathological hallmark of AD and several genes were found in GWASs:
CD33MEF2cHLA-DRB5/HLA-DRB1CR1MS4AABCA7TREM2JNPP5DCLUEPHA1
3. Endocytosis:
PICALMCDZAPBIN1SORL1

Cholesterol metabolism is involved in the pathogenesis of AD. High cholesterol levels in midlife increase the risk of AD [107] and ApoE protein is the major cholesterol carrier. Endocytosis is a critical process in synaptic transmission and response to neural damage. Several genes were found in GWASs, most of them are involved in APP trafficking that plays a key role in AD pathogenesis.

Recent meta-analysis of GWASs has identified additional genes implicated in LOAD: CASS4, PTK2B, NME8, ZCWPW1, CELF1, FERMT2, SLC24A4/RIN3 and DSG2. Functions of these genes are yet poorly characterized.

Advances in sequencing technologies (whole-exome and whole-genome sequencing) resulted in identification of novel genes associated with both EOAD and LOAD. The most important novel genes are: PLD3, UNC5C, AKAP9 and ADAM 10.

Very recent studies have characterized the genetic networks associated with AD from large sampling of postmortem brain tissues [108]. Analysis of routes of possible disease initiation processes uncovered a number of deregulated biological processes and pathways (e.g., neuron differentiation, aging, oxidative stress, chemokine signaling, LTP, etc.)

In summary, AD is a complex neurodegenerative disease with a strong genetic component. With the advance of molecular genetics, linkage studies revealed APP, PSEN1, PSEN2 and APOE as AD genes. GWASs have identified over 20 loci associated with AD risk. These results may help in the development of effective prevention and treatment strategies for AD.

## 5. Effect of Lipids in APP Processing and the Role of APP Metabolites in Lipid Homeostasis

There is a close connection between AD related proteins and lipid metabolism. Several lipid classes and fatty acids have been found to be altered in AD brains. It was demonstrated that Aβ and AICD regulate several lipid metabolism pathways. Inversely, APP processing and thus formation of Aβ and AICD is strongly influenced by the surrounding lipid environment, indicating a bidirectional link between APP proteolysis and lipid metabolism.

The link between AD pathology and lipid homeostasis was strengthened by the discovery that the APOE gene ε4 allele is the strongest genetic risk factor for LOAD. ApoE is the major cholesterol carrier, which influences Aβ clearance, aggregation and deposition in an isoform–dependent manner. Studies suggest that ApoE4 protein is less efficient than ApoE2 and ApoE3 in Aβ clearance [109]. APOE ε2 allele suppresses the initiation of AD development by lowering the cholesterol levels in synaptic membranes [110].

The surrounding lipid bilayer has strong impact on APP-processing. Increased membrane fluidity may stimulate the non-amyloidogenic APP-processing by reducing APP internalization [111]. The generation of Aβ mainly takes place in lipid rafts, membrane microdomains enriched with cholesterol, gangliosides and other sphingolipids, where APP and the β- and γ-secretases are together. (The non-amyloidogenic APP-cleavage occurs predominantly in non-raft-regions [112].) Modulation of the membrane lipid composition might influence Aβ-generation. Most recently, the bidirectional link and regulatory feedback cycles between APP and its metabolites as well as lipids have been widely reviewed [113,114]. The most important discoveries are:

1. *Cholesterol*: A mutual regulatory feedback cycle exists, in which cholesterol influences APP processing to Aβ, while de novo, cholesterol synthesis is inhibited by high level of Aβ. Modification of cholesterol levels has effects on multiple proteins, not only APP and Aβ [115].
2. *Docosahexaenoic acid* (*DHA*) is a polyunsaturated fatty acid. There are several epidemiological and experimental indications for a beneficial effects of DHA in preventing AD, at least at very early disease stages.
3. *Plasmalogens* (*PLs*) represent 22% of the total phospholipid mass in human brain tissue. In AD pathogenesis a vicious circle between PLs and Aβ-generation might be postulated. Accumulated Aβ reduces cerebral PL contact and that stimulates γ-secretase activity, which leads to further Aβ-production [114].
4. *Sphingolipid* (*SL*) impact on AD. Sphingolipids modulate APP-processing and Aβ-aggregation and, inversely, AICD downregulates total sphingolipid biosynthesis [116]. Sphingolipids are major components of lipid raft and the different SL classes have impact in AD-pathogenesis:
  *Ceramide* is accumulated in AD-patients. A feed-forward cycle between ceramide and Aβ exists in AD brain: increased ceramide level leads to enhanced Aβ production and that elevates ceramide synthesis, which stimulates Aβ-production. *Sphingosine* content elevates during the progress of AD [117]. In contrast, cerebral *sphingosine-1-phosphate* (*S1P*) content declines in AD patients, and negatively correlates with the level of Aβ.
  *Gangliosides*, sialic acid containing glycosphingolipids represent a heterogeneous group of different subtypes (GM1, GM1a, GD1b, GT1b GM3, etc.). There is strong link between ganglioside homeostasis and AD, however, further studies are needed to identify the most promising molecular target for AD therapeutic approaches.

In summary, several sphingolipid classes have been shown to affect the proteolytic processing of APP and Aβ-clearance: ceramides and total gangliosides. GM1 and GD3 are associated with an increased Aβ level, while sphingomyelin and GM3 have the opposite effect.

## 6. Dysregulation of Protein Homeostasis in AD

**Figure 2:**
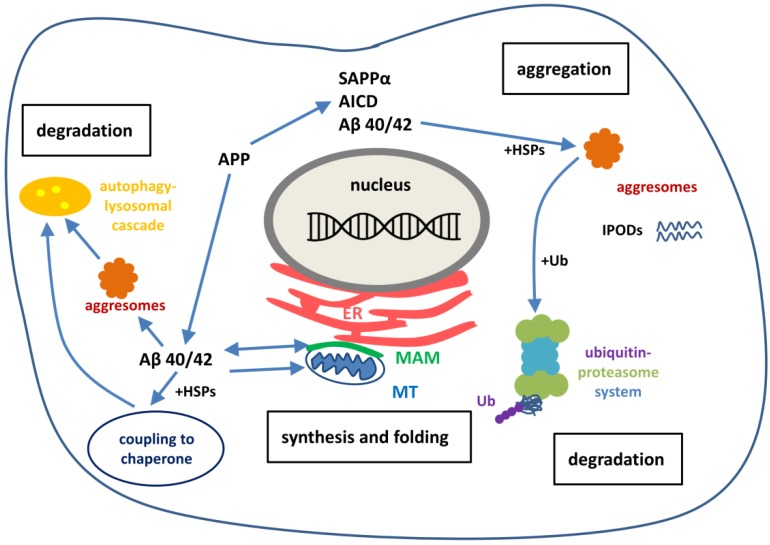
Schematic representation of the intracellular proteostasis network. Cytosolic misfolded proteins are bound by molecular chaperones, such as the HSPs. Molecular chaperones may act as aggregate unfolding and refolding enzymes. Autophagy pathways (chaperone-mediated, micro- and macroautophagy) degrade misfolded proteins. Ubiquitinated proteins are degraded by the ubiquitin-proteasome system. Sequestration of degraded misfolded proteins may form compact aggresomes and IPODs. Proteasome resistant aggresomes become enveloped by autophagosome membranes and transported into the lysosomes for autophagy.

During the last ten years extensive research has been performed for studying the pathomechanism of NDDs. These studies suggest that the first step of initiation of AD and other NDDs is not the accumulation of misfolded protein aggregates in neurons, but rather the severe dysregulation of protein homeostasis. NDDs share common cellular and molecular pathological mechanisms, such as excitotoxicity, calcium dysregulation, oxidative stress, mitochondrial dysfunction, ER stress and the failure of the protective processes for removal of misfolded proteins. The term protein homeostasis (proteostasis) refers to the maintenance of all proteins in the proteome in a conformation, concentration and location that is required for their correct functions [118]. An extensive and complex network of signaling pathways are involved in safeguarding cells and organism against proteotoxic stress [119]. The task of the system is to maintain the balance between the production and disposal of proteins. The proteostasis network contains pathways that regulate the biogenesis, folding, trafficking and degradation of proteins (Figure 2).

The removal of toxic forms of misfolded proteins by aggresomes, autophagy or the ubiquitin-proteasome system (UPS) is especially important in post-mitotic cells, such as neurons, since these cannot be readily replaced. In neurons, the maintenance of proteostasis plays a key role to healthy aging, dysregulation of the network can lead to NDD. APP, SAPPα and Aβ peptides are involved at the onset of AD.

### 6.1. The Double Edged Sword: Neurotrophic APP as A Source of Toxic Aβ

Aggregation-prone proteins play important roles in the onset of NDDs. APP itself and its soluble extracellular fragment SAPPα are not aggregation-prone and act as protective factors in acute neuronal insults [37,38,39]. APP, as a neuroprotective protein, may play a key role in neuronal homeostasis, very probably by regulation of Ca^2+^-homeostasis (see Section 2). APP biosynthesis is up-regulated for neuronal survival in acute and chronic stress situations. In an unexpected manner, this mechanism may increase the risk of formation of the aggregation-prone Aβ by processing of APP by β- and γ-secretase.

Analysis of the genetic background of EOAD demonstrated that several mutations of one gene (APP, PSEN-1 or PSEN-2) are satisfactory for manifestation of EOAD owing to continuous formation of toxic Aβ assemblies. The onset of the disease is relatively early (age of 45–65 years, see Section 4), toxic Aβ initiates the pathogenesis.

LOAD is a polygenic disease, and thus also environmental factors are needed for its manifestation beyond the genetic background. Aging is the key risk factor due to the progressive decline of protein homeostatis. In early life, the overexpression of aggregation-prone proteins in the cell can be balanced by the proteostasis network. The capacity of this network declines during aging [8]. (An example: age-related declination of heat-shock factor 1 (HSF1) decreases the activity of chaperones.) Different cellular stress conditions result in APP overexpression in aging brain. Although, the protein itself is neuroprotective, chronic up-regulation of APP may result in the formation of Aβ and neurotoxic Aβ aggregates. A simultaneous reduction or dysfunction of proteostasis capacity during aging results in an accumulation of toxic Aβ aggregates and thus the onset of LOAD. To summarize our present knowledge, APP might play an important role in the pathomechanism of both EOAD and LOAD: APP represents the source of aggregation prone Aβ peptides.

### 6.2. Formation of Intracellular Aβ in Neurons

For many years, extracellular Aβ (eAβ, particularly eAβ 1-42) has been thought to be one of the primary causes of AD [15,120], mainly because of the extracellular location of senile plaques. This hypothesis was supported by the facts that fibrillar and oligomeric Aβ could be toxic to neurons, activate microglial inflammation, induce Tau-hyperphosphorylation and directly damage membranes. Recent publications demonstrate unequivocally that neuronal (also neurotransmitter) receptors are targets of eAβ. Strong evidence supports that water-soluble, oligomeric eAβ-triggered aberrant signaling may cause cognitive dysfunction and consequent neurodegeneration in the brain [121,122,123,124].

Experimental studies demonstrated that two pools of Aβ exist in the brain: extracellular and intracellular, and a dynamic relationship exists between them. More and more evidence suggests that eAβ may have a reduced impact on AD pathology, and eAβ deposition alone cannot be responsible for the whole AD pathology. The “intracellular Aβ hypothesis” is becoming more and more accepted [125]. This hypothesis assumes that iAβ accumulation is the early sign of AD and a causative event in disease development. iAβ is a cytotoxic substance and the eAβ deposition (in the later stage of AD) is rather the result of cell death and destruction. Mechanism of amyloid plaque formation also suggests an intracellular basis of Aβ pathogenicity [126].

A series of review articles has summarized the mechanism of iAβ accumulation and the results of iAβ experiments performed during the last ten years [125,127,128,129,130,131,132]. iAβ has been widely detected in neuronal cells and primary human neurons, and has a very broad range of interactions with cell organelles and proteins [133,134,135,136,137]. Very recently, intraneuronal accumulation of Aβ was observed by light and fluorescent microscopy as well as immune-electron microscopy [138]. Studies in several mouse models of AD with intraneuronal expression and accumulation of Aβ have proven that iAβ causes the onset of early AD related cognitive deficits in transgenic mice [139,140,141,142].

The intraneuronal pool of Aβ has a double origin: slow production of iAβ from APP inside the neurons [127,128,129,130,131,143,144,145] and uptake from the extracellular space. iAβ may induce subcellular compartment structural changes, e.g., stable expression of human iAβ increases the number of Golgi apparatus elements, lysosomes and lipofuscin bodies in the hippocampus in a double APPxPS1 mutant transgenic rat model [146]. iAβ is selectively resistant to degradation and accumulates in lysosomes. This lysosomal accumulation is mechanistically similar to prion replication in a cyclic process [147,148]. Accumulation of iAβ takes place in the solid phase and forms a heterogeneous population and results in nonfibrillary iAβ-species [149]. Aberrant iAβ accumulation begins in endosomes. The endosomal-lysosomal (EL) system is one of the most important sites of Aβ metabolism. Insoluble iAβ, resistant against proteases, accumulates in the EL-system and then the iAβ aggregates trigger neuronal death [150,151]. Aβ accumulation in the EL system (sometimes called late endosome/multivesicular bodies, MVBs) increases lysosomal membrane permeability and causes the release of the lysosomal content (cathepsins) into the cytoplasm starting apoptotic cell death. One of the earliest events in iAβ-mediated in vitro neurotoxicity is just the release of lysosomal proteases [152].

In summary, a series of experiments demonstrated the formation and accumulation of iAβ, including the observation of iAβ by fluorescent and immune-electron microscopy. The presence of iAβ in the neurons results in interaction with cellular proteins and subcellular organelles.

### 6.3. Intracellular Aβ Affects the Function of Subcellular Organelles: Ca^2+^ Dysregulation, the ER-Mithochondria Axis and Cross-Talk

Proof of mitochondrial localization of iAβ is an important result in AD research. Mitochondria play a key role in energy metabolism, their role in aging and AD pathogenesis has been emphasized for years. Mitochondrial Aβ may have a decisive role in the pathological cascade leading to neuronal dysfunction and AD pathology [153,154,155]. It was found that iAβ affects mitochondrial failure and dysfunction. Brain hypometabolism and progressive decrease of brain metabolism were early signs of AD and other NDDs [34,156,157,158,159]. AD-related hypometabolism is linked with regional vulnerability of neurons in AD-brain [160]. The presence of mitochondrial Aβ correlates well with mitochondrial dysfunction in the synapses [161]. Changes in the level of individual mitochondrial proteins could serve as early metabolic markers for AD [162]. Aβ was found to impair the import of mithochondrial pre-proteins within the organelle, because of its aggregation with these proteins [163,164].

The endoplasmic reticulum (ER), a dynamic multifunctional organelle, plays a decisive role in Ca^2+^ homeostasis, protein folding and transportation and neuronal death. ER is responsible for protein assembly as well as for post-translational modifications of the peptide chains (e.g., glycosylation, disulfide bond formation, etc.). ER also monitors the quality of nascent proteins.

**Figure 3:**
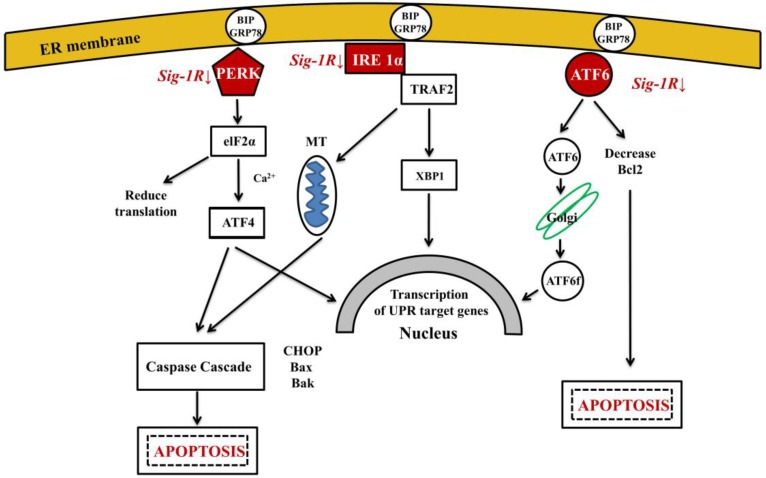
Three signal pathways of ER-stress activate UPR. ER chaperone GRP78 under normal conditions binds all the three ER-stress sensors (PERK: protein kinase RNA like ER-kinase; IRE1α: inositol requiring enzyme 1α; ATF6: activating transcription factor 6). Under ER-stress GRP78 dissociates from sensors. PERK and IRE1α will be phosphorylated and oligomerized, ATF6 translocates to the Golgi (Abbreviations: eIF2α: eukaryotic translation initiation factor 2α; XBP1: X-box binding protein 1 (spliced form); TRAF2: TNF-associated factor-2; ATF4: transcriptional activator factor-4; MT: mitochondrium). ATF6, ATF4 and XBP1 activate UPR target genes to enhance the capacity of the ER to cope with unfolded proteins. Activation of Sig-1R inhibits the three branches of UPR.

Several factors, including ER Ca^2+^-depletion, metabolic disturbances, oxidative stress and trafficking of proteins through the secretory pathway may initiate misfolded protein accumulation in ER lumen and lead to ER–stress. The possible involvement of ER-stress in the pathogenesis of AD has been studied [165]. It has been shown that ER-stress enhances γ-secretase activity by up-regulating PSEN1 levels and thus it might be involved in Aβ-formation and in the initiation of AD [166]. When cells are exposed to stressful conditions that perturb ER function, unfolded proteins accumulate in ER lumen. For maintaining and re-establishing protein homeostasis, ER activates the unfolded protein response (UPR) [167,168,169]. Three signaling pathways operate in parallel to sense ER-stress and activate UPR: IRE1α, PERK and ATF6 [34] (Figure 3). UPR is a bi-functional cellular response to misfolding, having both pro-survival and pro-apoptotic effects. Ca^2+^ regulates cell death both at the early and late stages of apoptosis, and severe Ca^2+^ dysregulation can promote apoptotic cell death [170]. Recently, the UPR theory has been demonstrated in a *C. elegans* model of AD: basal activity of the UPR was beneficial under normal conditions, however, inducible Aβ peptide expression caused repression of the signaling [171].

Very recent experimental results have validated the old Ca^2+^ hypothesis of AD: intracellular Ca^2+^ (iCa^2+^) plays a key role in the pathomechanism of AD. Dysregulation of iCa^2+^ homeostasis has been suggested as a common proximal cause of neural dysfunction [172,173]. Also targeting Ca^2+^-signaling pathways has been proposed as a novel therapeutic possibility to preserve cognitive functions in AD [174,175]. iCa^2+^ levels are tightly regulated within a narrow physiological range. Ca^2+^ enters the cells via different Ca^2+^-channels (receptor-operated, voltage dependent, store operated channels and sodium/calcium exchanger). Calcium may also be released into the cytoplasm from the ER through inositol-1,4,5-triphosphate (IP_3_R) and ryanodine receptors (RyRs). Different systems counterbalance the iCa^2+^ increase in the cell: plasma membrane Ca^2+^-pump and the sarcoendoplasmic reticulum ATP-ase (SERCA) restore physiological Ca^2+^-levels. Mitochondria play a central role also in Ca^2+^ buffering and apoptotic signaling. The excess of iCa^2+^ can be taken up by mitochondria through the mitochondrial Ca^2+^-uniporter (MCU) [176,177].

ER-to-mitochondria contacts represent important structures linked to multiple cell processes, such as Ca^2+^-signaling, UPR, proliferation and cell death, inflammation, lipid metabolism and autophagy [178]. The ER-mitochondria axis is progressively emerging as a complex signaling platform for cell survival [179,180]. This axis controls cellular Ca^2+^-homeostatis and thus the cellular metabolism, by regulating mitochondrial activity and the action of different hormones. A sub-region of the ER, referred to as MAM (Section 2), is physically and biochemically in contact with the outer mitochondrial membrane. MAM is an inter-organelle interface, its proteins connect the ER to mitochondria. MAMs are intracellular lipid rafts that regulate Ca^2+^-homeostasis and glucose-, phospholipid- and cholesterol metabolism, maintenance of membrane-lipid composition [181,182]. All of these processes are altered in AD.

MAM structure and function as well as ER-mitochondrial interplay have been extensively studied. A series of MAM-associated proteins have been characterized [183,184]. The most important proteins are:

- phosphatidylserine synthase-1 (PSS1)
- fatty acid EoA ligase 4 (FACL4)
- inositol -1,4,5-triphosphate receptors (IP3Rs)
- voltage-dependent anion channel (VDAC1)
- sigma-1 receptor (Sig-1R)

ER-mitochondria Ca^2+^-transfer is the central process of the communication between the two organelles. A constitutive IP3-receptor mediated ER-MT Ca^2+^-transfer stimulates mitochondrial respiration, increases ATP production and guarantees normal cell bioenergetics [185]. The PSS1 protein is in the ER-side of MAM, it is essential for the transfer of Ca^2+^ from the ER at MAM [184]. VDAC1 is localized in the mitochondrial site of MAM and forms bridging complexes for Ca^2+^ transport (e.g., IP3R 1-VDAC1) [186]. The details of the processes have been recently reviewed [187].

AD patients display different early intracellular alteration, such as Ca^2+^ and lipid dyshomeostasis, increased ROS-levels, impairments in autophagy, axonal transport and mitochondrial dynamics. MAM is the central hub of these activities [178] and might be the place of APP-processing and iAβ production (see Section 2). In vitro studies demonstrated that APP 695 accumulates in the ER upon ER-stress [188]. Katayama et al. [189] found that the loss or dysregulation of UPR can initiate neuronal degeneration through Aβ generation as a consequence of APP processing. It has been proposed that MAM alterations (e.g., upregulation) are the background of LOAD [190]. Pathogenic level of Aβ strengthens ER-to-mitochondria Ca^2+^-transfer [191] and an increased ER-mitochondria coupling has been observed in different AD models [191].

In summary, iAβ might be localized in the mitochondria, affecting mitochondrial failure and dysfunction. iAβ disturbs the ER-mitochondrial interplay, the ER-to-mitochondria Ca^2+^-transfer and thus cellular Ca^2+^-homeostasis.

### 6.4. Molecular Chaperones in AD

Molecular chaperones belong to the most important factors in proteostasis. They form the first line of defence of cells against protein misfolding and subsequent aggregation [192]. Analysis of the human intracellular “chaperome” identified 332 chaperone and co-chaperone genes that were placed into nine families [193]. Five of these chaperone families correspond to heat-shock proteins (HSPs: HSP90, HSP70, HSP60, HSP40 and small HSPs.). Chronic proteotoxic stress results in the induction of the heat shock response (HSR) by the activation of the transcription factor HSF1. This activation leads to the rapid expression of the HSP molecular chaperones [194]. HSPs play various roles in most cellular processes e.g., they regulate stress responses, traffic proteins, buffer apoptotic signalling, shuttle misfolded proteins for degradation by the proteasome or by autophagy. The roles of individual HSPs in proteostatis are widely reviewed [195,196,197].

Human brain chaperone levels decrease during aging, and cellular senescence of neurons further decreases chaperone activity with age and in NDDs. Therapeutic application or up-regulation of HSPs represent novel possibilities for treatment of NDDs. Overexpression of individual HSPs can be an over-simplification owing to the interdependence of chaperones and compensatory mechanisms within the chaperome. As an example, the chaperone-meditated degradation of aggregation-prone proteins by autophagy required the concerted actions of HSP70 and HSP40 as well as two co-chaperones Hip and Hop [198]. Overexpression of HSP70 in mice could prevent the aggregation of Aβ and the associated toxicity [199]. It was found that Aβ was co-localized with HSPB8 in AD brain [200]. Sequestering proteins into chaperone-enriched aggregates prevented an age-related decline in proteostasis and prolonged lifespan in *C. elegans* [201]. HSPs very probably inhibit aggregate fragmentation to cytotoxic oligomers [202] and facilitate degradation of the aggregates by chaperone-mediated autophagy. A complex chaperon network in ER lumen orchestrates the secretory protein folding [203]. This network ensures proper folding of native proteins in a crowded cellular environment accomplishing highly efficient production of the secretome [204]. Glucose regulated protein 78 (BiP or GRP78) and calreticulin (CRP55) as chaperones of the HSP family stabilize the cell. Sig-1R, a molecular chaperone is also a cytoprotective protein by participating in Ca^2+^-regulation at MAM. Sig-1R helps Ca^2+^-homeostasis by stabilizing IP3 receptor type 3 (IP3R3) [184]. Sig-1R agonists have been used in the therapy of neuropsychiatric diseases (reviewed in [205]).

To summarize these studies, HSP-s play a crucial role in proteostasis. The levels of HSPs decrease during aging and thus formation of misfolded proteins is increased. Simultaneously, HSP-dependent degradation and clearance of amyloid is decreased resulting in a growing imbalance in the mechanism of proteostasis.

### 6.5. Aggresome Activity for Maintaining Proteostasis

Protein aggregation and the formation of inclusions are closely associated with neuronal degeneration [206]. Aggregation alone provides strong evidence that proteostasis has been disrupted in these diseases. We are only just beginning to understand the complex cellular processes that actively promote the formation of inclusions in cells. It is still not clear that formation of a protein inclusion is a cause or a consequence of NDDs, but it may be detrimental to suppress this process with anti-aggregating small molecules [119].

It is now clear that cells are able to actively sequester misfolded and aggregating proteins into subcellular compartments. (These compartments are not organelles but spatially distinct regions in the cell.) This compartmentalization minimizes the risk that misfolded proteins pose to cells (e.g., nucleation of further proteins) and opens the door for degradation or resolubilization. Several mechanisms for formation of inclusions have been described in the cell:

1. Formation of aggresomes or aggresome-like structures [207]. In aggresomes the sequestered proteins are associated with chaperones and proteasome subunits [208].
2. Insoluble protein deposits (IPODs): the proteins within are not ubiquitinated and mostly form amyloid fibrils [209].
3. Juxtanuclear qualitity control (JUNQ) [209] inclusions contain ubiquitinated proteins, proteasome subunits and chaperones (e.g., HSP70). JUNQ was proposed to be a cellular quality control center in which soluble misfolded proteins or aggregates from the cytosol accumulate for proteosomal degradation and refolding [209].

### 6.6. Balancing Proteostasis by Protein Degradation

The ubiquitin-proteasome system (UPS) is the primary selective system in eukaryotic cells, a precise mechanism that maintains the proper concentration of many regulatory proteins of the cell. In addition, UPS is a key component of the proteostasis network to terminate damaged proteins [210]. The UPS degrades more than 80% of normal and abnormal, misfolded intracellular proteins [211].

Ubiquitination is one of the most abundant posttranslational modification in cellular signaling that regulates a broad range of cellular pathways. Ubiquitin (Ub) labels substrate proteins via a highly ordered multi-step enzymatic cascade. In the CNS, Ub contributes to neuronal growth and development, excitability, neurotransmission, long term potentiation as well as synapse formation and elimination [212]. Maintenance of the Ub-proteasome system is central to neuronal health, as neurons are very sensitive to prolonged Ub deficiency which leads to cell death [213]. The ubiquitination machinery is a highly promising target for human therapy [213,214,215] as much evidence supports the role of compromised Ub-homeostasis in the pathophysiology of NDDs. Probably the most important evidence for the role of Ub in NDDs came from the widely observed enrichment of Ub in cytoplasmic inclusion bodies of many NDDs. Studies on genetically modified, Ub deficient mice demonstrate that the maintenance of Ub homeostatis is necessary for neuronal protection [216].

Components of the Ub-proteasome system are very important potential targets in AD management. The recent therapeutic developments are summarized in [214,215].

The autophagy-lysosome system. The broad term autophagy is a process by which cytoplasmic constituents (e.g., organelles, macromolecules) are degraded by the lysosome [215,217]. Molecular definitions and different subtypes of autophagy were reviewed in 2017 [218]. Autophagy involves the formation of double membrane cytosolic vesicles (known as phagosomes), which transport the engulfed long-lived proteasome resistant proteins and particular organelles (e.g., mitochondria) to the lysosomes for degradation [219]. Failure of each form of autophagy (microautophagy and endosomal microautophagy, chaperone mediated autophagy, macroautophagy) may play an important role in the initiation of NDDs. Mutations of genes regulating autophagy cause NDDs, such as AD, across the age spectrum with exceptional frequency [220]. Recent GWASs suggested that compromised autophagic-lysosomal mechanisms underlie (and thus contribute to) NDDs. Lysosomal pH is acidic and defective lysosomal acidification might cause autophagy failure and thus AD [221]. Numerous studies show lysosomal dysfuntion in AD, including perturbed trafficking of lysosomal enzymes and, as a consequence, accumulation of APP metabolites. This evidence leads to the hypothesis that endo-lysosomal and autophagic dysfuntion might be a driving factor for AD [222]. Autophagy appears to be mechanistically linked to disease progression in AD [223]. Autophagy vacuoles are abundant in AD neurons and contain substantial amounts of Aβ [224]. Increased autophagy compartments occur at early stages of the disease, when amyloid deposits are not visible. However, autophagy declines with the disease progression. Both the maturation and transport of autophagosomes are impaired and thus, Aβ-containing autophagosomes appear in AD [224,225]. Impaired lysosomal proteolysis may also contribute to Aβ accumulation. Recent studies focuses on the relationship between aging and autophagy gave interesting results [226,227]: senescence of cells was associated with decreased autophagy, decreased expression of autophagy genes. It occurred as the result of the increased methylation of autophagy genes by the DNA methyl transferase DNMT23. Researchers identified the gene encoding ATG5, an autophagy protein involved in the elongation of autophagosomes, as one of the highly methylated autophagy genes. Evidence demonstrates the role of both cytoplasmic and nuclear mechanisms in the regulation of autophagy [228].

In summary, different protein degradation systems (UPS, several autophagy pathways) serve to maintain proteostasis in cells. There is a great hope that UPS and the autophagy-lysosomal cascade may provide therapeutic targets for the treatments of NDDs, among them in AD [229]. Up-regulation of degradative pathways might be a therapeutic method, however, many unknown factors should still be studied before therapeutic application [119,220].

## 7. Vascular Risk Factors: The Role of the Neurovascular Unit and the BBB in AD

Aβ clearance from the brain is mediated by various mechanisms, such as phagocytosis by glial cells, enzymatic degradation, transport to the cerebrospinal fluid (CSF) with subsequent re-absorption into the venous circulation and direct transport across the BBB [230].

It is well-known that chronic cerebral hypoperfusion and BBB dysfunction could precede the manifestation of AD by years or even decades [231]. Hypoxia activates the amyloidogenic processing of APP leading to Aβ accumulation in the brain [19]. Vascular dysfunction can provoke AD pathology by activating β- and γ-secretases.

The neurovascular unit (NVU) includes clusters of glial cells, neurons and pericytes and undergoes functional changes during aging [232]. These changes may contribute to neuronal injury and cognitive deficit. The BBB is formed by the capillary endothelium, the basement membrane, and the surrounding pericytes and astrocyte end-feet. The BBB operates within the NVU. The role of vascular dysfunction in the pathogenesis of AD has recently been reviewed [233].

Much evidence demonstrates that age and cardiovascular conditions initiate cerebral hypoperfusion (CCH) that increases the risk of AD. Chronic CCH causes BBB dysfunction [233]. The BBB may have fundamental, even though causal role in AD [234]. Three mechanisms are considered to participate in the initiation of the disease: decreased bulk flow, decreased BBB clearance of Aβ and decreased insulin transport:

1. Decreased bulk flow occurs because of reduced production of CSF. With aging (and even more so in AD) the reabsorption of CSF back into the circulation is reduced [235]. CSF levels of neurotoxic substances, such as Aβ increase, this process is further aggravated by a slowing of the glymphatic circulation [234,236] and BBB disruption. Neuroinflammation might be the background of these changes.
2. In AD, the BBB undergoes functional and structural changes that disrupt its gate function, impair energy supply to the brain, reduce the clearance of Aβ and produce neurotoxic molecules. Decreased BBB clearance of Aβ leads to an increased amyloid burden in the brain and the initiation of AD [237]. The brain-to-blood efflux of Aβ is mediated by the lipoprotein receptor related protein (LRP-1), and the ABC subfamily B member 1, also termed P-glycoprotein (P-gp). Data from mouse models suggest that LRP-1 and P-gp expressions decline with age [238] suggesting a possible path for increased Aβ deposition and decreased clearance. Patients with AD show decreased levels of LRP-1 and P-gp [239] in BBB cells, leading to increased oxidation of LRP-1 and decreased level of P-gp activity. Knockdown of LRP-1 in mice recapitulates the key features of AD predicted by the neurovascular hypothesis (decreased Aβ-clearance, increased brain levels of Aβ and impaired cognition). Once initiated, decreased clearance of Aβ can further impair LRP-1 function by oxidation. Decreasing Aβ levels in animal models of AD restores Aβ clearance and cognition [240]. Inflammation also impairs Aβ-clearance from the brain and increases pericyte uptake of Aβ. This mechanism may account for the loss of pericytes seen in AD [241]. Taken together, BBB impairment can promote or even initiate AD: BBB dysfunction causes oxidative stress. Inflammation and mitochondrial dysfunction can mediate a vicious circle causing neuronal damage and increase of Aβ level in the brain [233]. BBB-mediated Aβ accumulation is predominantly based on reduced efflux across the barrier.
3. The brain is dependent on BBB transport of insulin; this hormone has neurotrophic and neuroprotective functions in the CNS. In AD, the transport of insulin across the BBB is decreased. Such a transport defect reinforces that insulin resistance occurs in the brain of AD patients [242]. In clinical studies, delivery of insulin to CNS could improve some aspects of cognition of AD patients within 15 min [243].

In summary, aging and cardiovascular conditions initiate cerebral hypoperfusion and BBB dysfunction that may mediate decreased Aβ clearance and the onset of AD.

## 8. AD as An Inflammatory Disease: The Role of Microglia

Neuroinflammation is one of the main triggers of neurodegeneration. The concept of linking AD to neuroinflammation emerged in the early 90s when it became evident that an altered immune response could be observed in AD. Neuroinflammation is primarily driven by microglia, perivascular myeloid cells and astrocytes, acting as triggers for AD pathogenesis either independently or in combination with the effects of Aβ [244]. Microglia and astrocytes are two major cell components of the CNS innate immune system. Microglia play a macrophage-like role in the immune defense. Microglia exerted neuroprotective activity by degrading Aβ in an Aβ overexpressing AD mouse model (APPxPS1) [245]. Microglia-mediated clearance of Aβ occurs through the TLR4 receptor. As Aβ is a TLR4 ligand, chronic exposure of the receptors to Aβ can result in TLR-signaling dysfunction and inflammation [246]. Aβ aggregates may also interact with other microglial receptors like CD14, CD36, CD47 and RAGE. Inflammasome activation occurs through the binding of Aβ to CD36 [247]. Inflammasomes link vascular disease with neuroinflammation and brain disorders [248]. Microglia regularly monitor their environment and regulate tissue homeostasis through the scavenging functions. Resident microglia have functions related to immune surveillance [249], adult neurogenesis and refinement of synaptic network by synapse pruning [250]. Contrary, novel experiments have demonstrated that microglia mediate synaptic loss in an early-AD mouse model [251]. Aβ 1-40 dimer was injected into the brains of wild type mice and a severe synaptic loss was found in 72 hours and postsynapses contained C1q and C3 complement proteins. Blocking the complement pathway (e.g., with anti-C1q antibodies) prevented synaptic loss.

In summary, microglia are a double edged sword playing double role in the onset of AD. Several experimental and epidemiological findings have demonstrate that microglia play a crucial role in Aβ removal by reduction of Aβ plaques, and thus attenuating the disease. On the contrary, other reports propose the harmful role of the activated microglia in AD: Aβ stimulation increased the production of pro-inflammatory cytokines such as TNFα and IL1β [252]. Microglia cells are needed for the protection of neurons (the brain is unprotected against injuries without microglia). Selective inhibition of microglia to switch off the formation of pro-inflammatory cytokines would be a realistic way for attenuating neuroinflammation in AD brain.

## 9. Conclusions: Major Trends in AD Prevention and Drug Discovery

**Figure 4:**
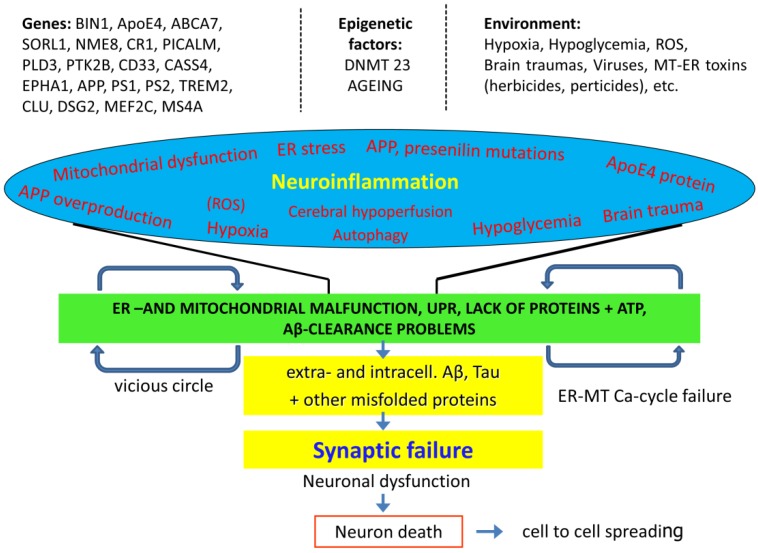
Genes of high and low penetrance, as well as epigenetic and environmental factors are participating in AD pathogenesis. Brain traumas, vascular problems (cerebral hypoperfusion, hypoxia, hypoglycemia), declining amyloid degradation and clearance with aging are involved in ER- and mitochondrial dysfunction and protein dyshomeostasis. Amyloid accumulation leads to neuroinflammation, synaptic failure, neuronal dysfunction and death.

The precise molecular mechanisms of AD are still not fully understood. The role of very important factors, such as APP and microglia is controversial in the initiation of AD. There is no breakthrough in the development of AD drugs [253]. However, it is clear that AD is a multifactoral disease with complex genetic background, and protein dyshomeostasis and aging are the main risk factors. Figure 4 shows the complexity of AD.

The main problem of the initiation and progression of AD is the dysregulation and imbalance of the proteostasis network in aging. It occurs years or decades before AD is manifested. We know already the sequence of changes that occurs during the asymptomatic phase of AD.

There is a great progress towards the understanding of the structural and physical properties of the amyloid state, the kinetics and mechanism of its formation, and the nature and origin of its links with the disease. We should understand the nature of protein homeostasis in healthy cells as well as the effect of suppression or inhibition of protein aggregation and amyloid formation to the pathogenesis of AD.

Over the last decade, more than 50 drug candidates have successfully passed phase II clinical trials, but none has successfully used in phase III [254]. Most of the drug candidates targeted different forms of Aβ with the aim of removing plaques or stopping them from developing. The failure of these trials changed the aim of AD drug research, the novel aims are prevention of the disease and “to stop Alzheimer’s before it starts” [253]. Prevention strategies [113,255] and new AD-drug targets have been outlined with this aim [119,254,256,257,258]:

1. Management of modifiable risk factors for dementia [255]. Beyond regular physical activity and decreasing cardiovascular risk, the nutritional factors are in the center of the research. Nutritional approaches have shown limited beneficial effects in clinical studies. The identification of apoE4 as the most prevalent genetic risk factor for AD emphasized the crucial role of lipids in the pathomechanism of AD (see Section 5). Based on the results of apoE and lipid experiments, novel methods are offered: different supplements can be combined in multinutrional approaches [113].
2. Development of compounds acting on the main stages of the pathogenesis of disease (the so called “disease modifying agents”). These drugs could potentially slow down the development of functional (and structural) abnormalities in the CNS, providing improvements of cognitive functions. Potential proteostatis-based therapeutics improving the imbalance between protein production and degradation belong to this group.
3. Focused design of multitargeted drugs acting on multiple molecular targets involved in the pathogenesis of Aβ. Some enzyme inhibitors, (e.g., Sig-1R agonists), anti-inflammatory agents and antioxidants are in these groups.
4. Repositioning of old drugs for new, anti-AD application offers a very attractive approach facilitating clinical trials. The oncolytic drugs bexarotene, tamibarotene, isotretinoin and long-acting insulin analogs belong to this group.

A number of radically new approaches for targeting key stages in AD pathogeneses were proposed and gave hope for successful development of novel therapeutics for AD treatments.

## Figures and Tables

**Table 1:** Examples for protein conformational, neurodegenerative diseases as well as their corresponding misfolded proteins.

List of Diseases	Misfolded Proteins
Alzheimer’s disease	β-amyloid (Aβ)
	hyperphosphorylated Tau (pTau)
	α-synuclein
	TDP-43
Parkinson’s disease	α-synuclein
Huntington disease	huntingtin
Lewy-body dementia	α-synuclein
Amyotrophic lateral scelerosis	TDP-43
Prion diseases	superoxide dismutase
	prion protein (PrP^sc^)

## Abbreviations

Aβ	β-amyloid peptide
AD	Alzheimer’s disease
AICD	APP intracellular domain
ALS	Amyotrophic lateral sclerosis
APP	Amyloid precursor protein
ATF 6	activating transcription factor 6
ATP	Adenosine triphosphate
BACE	Beta-site APP cleaving enzyme
Bak	Bcl-2 homologous antagonist killer
Bax	Bcl-2 like protein 4
BBB	Blood brain barrier
Bcl-2	B-cell lymphoma 2
BiP (GRP78)	Binding immunoglobulin protein
CCH	Chronic cerebral hypoperfusion
CNS	Central nervous system
CSF	Cerebrospinal fluid
DNMT	DNA methyl transferum
EOAD	Early onset AD
ER	Endoplasmic reticulum
ERAD	ER-associated degradation
GWAS	Genome wide association studies
HD	Huntington disease
HSP	heat shock protein
IRE1α	inositol requiring enzyme 1α
iAβ	Intracellular Aβ
IP_3_R	Inositol-triphosphate receptor
IL1β	Interleukin 1β
ID	Intrinsically disordered
IPOD	Insoluble protein deposits
JNK	c-Jun amino terminal kinase
LBD	Lewy body disease
LOAD	Late onset AD
LRP1	Lipoprotein receptor related protein-1
LTP	Long term potentiation
MAM	Mitochondrion associated membrane
MVB	Multivesicular body
NDD	Neurodegenerative disease
NVU	Neurovascular unit
PD	Parkinson disease
P-gp	P-glycoprotein
PERK	Protein kinase RNA like ER-kinase
PM	Plasma membrane
PSN1, PSN2	Presenilin 1 and 2
PrP	prion protein
ROS	Reactive oxygene species
SAPPα	Soluble APP-α
Sig-1R	Sigma1 receptor
SERCA	Sarcoendoplasmic reticulum ATP-ase
SOS	Superoxide dismutase
TLR4	Toll-like receptor 4
TBI	Traumatic brain injury
TGN	Trans Golgi network
TNFa	Tumor necrosis factor a
Ub	Ubiquitin
UPR	Unfolded protein response
UPS	Ubiqitin-proteasome system
